# Enhanced Photovoltaic Properties of Bulk Heterojunction Organic Photovoltaic Devices by an Addition of a Low Band Gap Conjugated Polymer

**DOI:** 10.3390/ma9120996

**Published:** 2016-12-08

**Authors:** Eui Jin Lee, Min Hee Choi, Doo Kyung Moon

**Affiliations:** Department of Materials Chemistry and Engineering, Konkuk University, 120 Neungdong-ro, Gwangjin-gu, Seoul 05029, Korea; fkaus21c@naver.com (E.J.L.); shain86@naver.com (M.H.C.)

**Keywords:** organic photovoltaics, ternary blend system, parallel-type bulk heterojunction solar cell, low band gap conjugated polymer

## Abstract

In this study, we fabricated organic photovoltaics (OPVs) by introducing the polymer additive HTh6BT into the photoactive layer of a poly(3-hexylthiophene):phenyl-C_61_-butyric acid methyl ester (P3HT:PCBM) system. The HTh6BT had a relatively low band gap energy of 1.65 eV and a molecular and crystalline structure similar to that of P3HT. In the photoactive layer, the HTh6BT and P3HT can both act as donors. In such parallel-type bulk heterojunctions, each donor can form excitons and generate charges while being separated from the donor/acceptor interface. Changes in the photovoltaic property of the OPV device by the addition of HTh6BT were evaluated, and the optical characteristics of the photoactive layer, as well as the surface morphology, polymer ordering, and crystallinity of the P3HT:PCBM film were analyzed. Compared to a device without HTh6BT, all short-circuit current densities, open-circuit voltages, and fill factors were enhanced, leading to the enhancement of the power conversion efficiency by 36%.

## 1. Introduction

Organic photovoltaic cells (OPVs) have several manufacturing advantages such as easy fabrication, low cost, flexibility, and high availability, and they are in the limelight as the next generation of solar cells for the production of clean and renewable energy [1,2]. OPVs with up to 10% power conversion efficiency (*PCE*) have been created through the design and synthesis of conjugated polymers (photoactive layer materials) [1,3,4], with efforts to develop green synthetic strategies [5,6], the introduction of additives for enhancing morphology control and charge carrier mobility [7,8,9], and energy barrier control through the introduction of an interlayer [10,11,12,13].

Generally, the maximum efficiency of a binary blend (polymer:fullerene) single-junction device is limited to 10%–12% due to the limited optical absorption and low charge transfer of the photoactive layer [14,15]. To overcome this limitation, researchers are attempting to develop tandem cells with a multilayer structure [16,17], as well as ternary blend OPVs [14,18,19,20,21,22,23]. However, tandem cells, despite the enhancement of the open-circuit voltage (*V_oc_*) [16,24] or the short-circuit current density (*J_sc_*) [24], show a slightly higher *PCE* than single-junction cells. Moreover, the production process is complex and expensive because multilayers are formed, and intermixing between layers must be overcome [25]. Compared to tandem cells, ternary blend OPVs can be manufactured more easily by adding donor materials to the existing binary blend solution. Depending on the material being added, the optical absorption characteristics of the photoactive layer can be controlled to enhance the efficiency [14,19,20]. Methods for manufacturing ternary blend OPVs include an expansion of the absorption range by mixing two polymers or small molecules with different absorption ranges [18,20,26], an improvement of the optical absorption and electron transfer characteristics by enhancing the ordering of the photoactive layer [19,27], and an enhancement of efficiency through energy transfer by matching photoluminescence (PL) characteristics with the absorption range of the host material [14,28].

Sharma et al. added a small molecule capable of absorbing light of long wavelengths (up to 700 nm) to the poly(3-hexylthiophen):phenyl-C_61_-butyric acid methyl ester (P3HT:PCBM) binary blend system, thereby enhancing the efficiency by expanding the absorption range of the photoactive layer [16]. Lim et al. introduced a low band gap material to the P3HT:PCBM system as an additive to expand the absorption range of the photoactive layer, and to enhance the ordering of P3HT [19]. Lu et al. applied a PID2 polymer additive to the PTB7:PC_71_BM or PTB7-Th:PC_71_BM system to enhance the optical absorption of the photoactive layer [14,28].

P3HT is a typical electron donor conjugated polymer used in bulk heterojunction OPVs; it yields a *PCE* of about 4%–5% when introduced to the binary blend system with PCBM [20,29,30]. However, because of its small absorption range (below 650 nm) and edge-on crystalline structure, P3HT is known to be inefficient for electron transfer in OPVs [31,32]. Sharma et al. expanded the absorption range by adding diketopyrrolopyrrole (DPP)-based conjugated small molecule sensitizers to the photoactive layer in order to enhance the *PCE* [20]. In such methods where a sensitizer is added, the energy level of the donor and/or acceptor should be considered in order to avoid the occurrence of exciton and charge traps [15]. In contrast, ternary or multi-blend systems of parallel type can enhance the *PCE* by adding a donor material with different band gap energies without considering the energy level. In such parallel-type bulk heterojunctions (PBHJs), each donor can form excitons and generate charges while being separated from each donor/acceptor interface [15,33].

In a previous study [3], poly[4-(4-hexyl-5-methylthiophen-2-yl)-7-(methylthiophen-2-yl)benzo[c][1,2,5]thiadiazole] (HTh6BT), a 3-hexylthiophene- and 2,1,3-benzothiadiazole-based polymer, was synthesized, and its characteristics were evaluated. The synthesized polymer, HTh6BT, had a band gap energy of 1.65 eV, which is low, and showed a π–π stacking distance (d_2_) of 3.9 Å, which is similar to that of P3HT (d_2_ = 3.8 Å) [3]. In this study, HTh6BT, which has a crystalline structure similar to the molecular structure of P3HT, was introduced as a polymer additive in the manufacturing of a PBHJ OPV. Changes in the photovoltaic property of the OPV device by the addition of HTh6BT were evaluated, and the optical characteristics of the photoactive layer, as well as the surface morphology, polymer ordering, and crystallinity of the P3HT:PCBM film were analyzed. Compared to a device without HTh6BT, all *J_sc_*, open-circuit voltages (*V_oc_*), and fill factor (*FF*) were enhanced, leading to the enhancement of the *PCE* by 36%.

## 2. Results and Discussion

**Figure 1:**
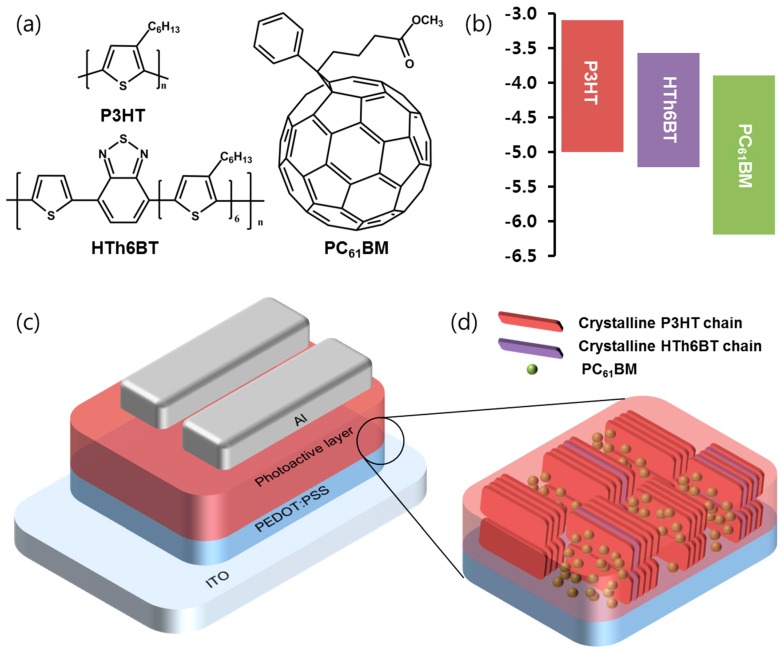
(**a**) Chemical structure of P3HT, HTh6BT, and PCBM; (**b**) Energy band diagram of P3HT, HTh6BT, and PCBM; (**c**) Device structure; (**d**) Illustration of the ternary blended active layer, in which the polymers induce the edge-on π–π stacking.

The chemical structures of the conjugated polymer P3HT, HTh6BT, and PCBM used in this study are shown in Figure 1. Their highest occupied molecular orbital (HOMO) and lowest unoccupied molecular orbital (LUMO) energy levels, as well as the fabricated device structure, are displayed. The energy levels of P3HT, HTh6BT, and PCBM refer to figures in the existing literature [3,18]. The HOMO and LUMO levels of HTh6BT are −5.22 eV and −3.57 eV, respectively. As shown in Figure 1b, they are positioned between the energy levels of P3HT and PCBM. Such an energy level alignment is able to induce an efficient separation of the photogenerated excitons from P3HT/HTh6BT, P3HT/PCBM, and HTh6BT/PCBM interfaces.

**Figure 2:**
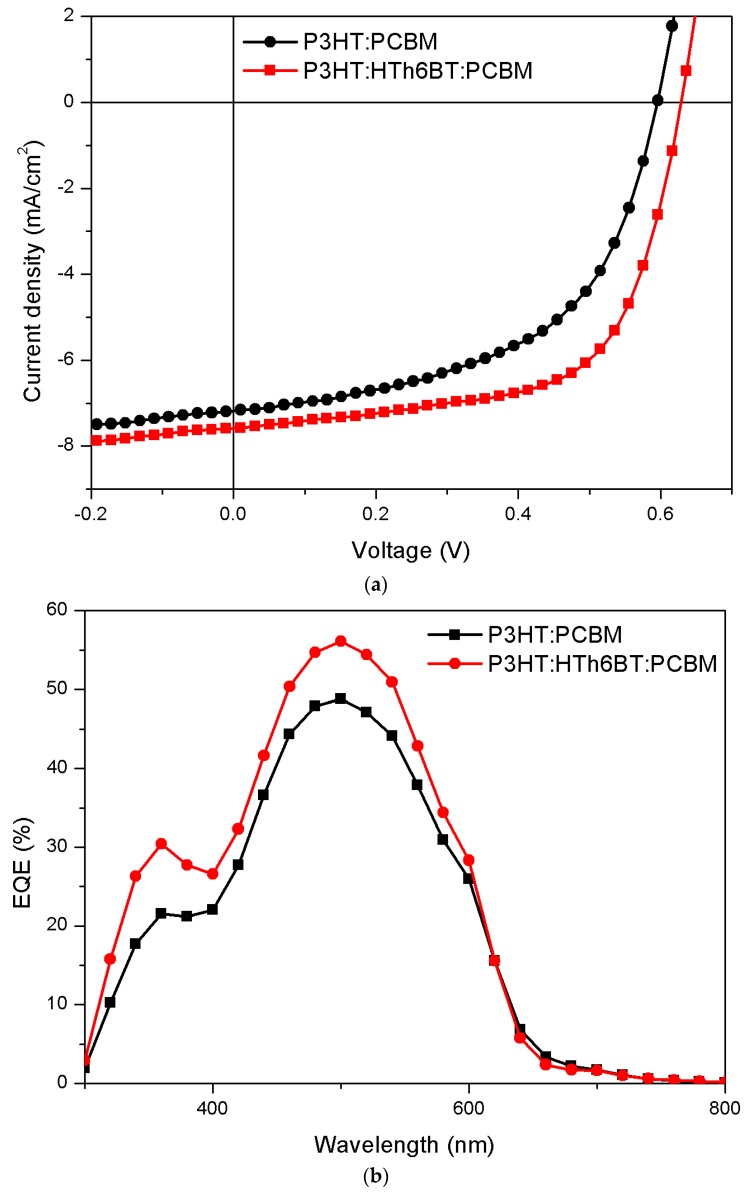
(**a**) *J–V* characteristics of polymer solar cells (PSCs); (**b**) External quantum efficiency (EQE) characteristics of PSCs.

When the weight ratio of P3HT:HTh6BT:PC_61_BM was 1:0.2:1, the photovoltaic performance of OPV devices was optimized (see Figures S1 and S2, Tables S1 and S2 in Supporting Information). Figure 2 shows the current density–voltage (*J*–*V*) curve, and the incident photon-to-current conversion efficiency (*IPCE*) data of optimized condition of device structure with ITO/PEDOT:PSS/P3HT:PCBM/Al and ITO/PEDOT:PSS/P3HT:HTh6BT:PCBM/Al, where ITO stands for indium tin oxide, PEDOT stands for poly(3,4-ethylene dioxythiophene), and PSS stands for poly(styrene sulfonate). Table 1 arranges the photovoltaic performances.

The device without HTh6BT shows *J_sc_*, *V_oc_*, *FF*, and **PCE** values of 7.1 mA/cm^2^, 0.596 V, 52.9%, and 2.2%, respectively. On the other hand, a PBHJ device with added HTh6BT shows enhanced performances of 7.6 mA/cm^2^, 0.636 V, 62.3%, and 3.0%, respectively. This indicates that a new pathway for generating and transferring charges can be created, and that the device efficiency can be enhanced by increasing the number of charges and by decreasing the chance of recombination, due to the addition of HTh6BT [34]. This is confirmed by the fact that the series resistance (*R_s_*) of the device decreased from 9.02 Ω·cm^2^ to 7.50 Ω·cm^2^, thereby increasing *J_sc_* from 7.1 to 7.6 mA/cm^2^, which eventually enhanced the *PCE*. In PBHJ OPVs, *V_oc_* is known to be determined by the mass fraction of the donor material [15]. In this study, the *V_oc_* of the PBHJ device was 0.636 V, which was enhanced when compared to the P3HT:PCBM device. This enhancement can be explained by the addition of the HTh6BT polymer, which shows a high *V_oc_* of 0.80 V in the HTh6BT:PCBM BHJ device. Based on this information, a PBHJ OPV is considered to have been successfully manufactured, wherein P3HT and HTh6BT polymers each play donor polymer roles.

**Figure 3:**
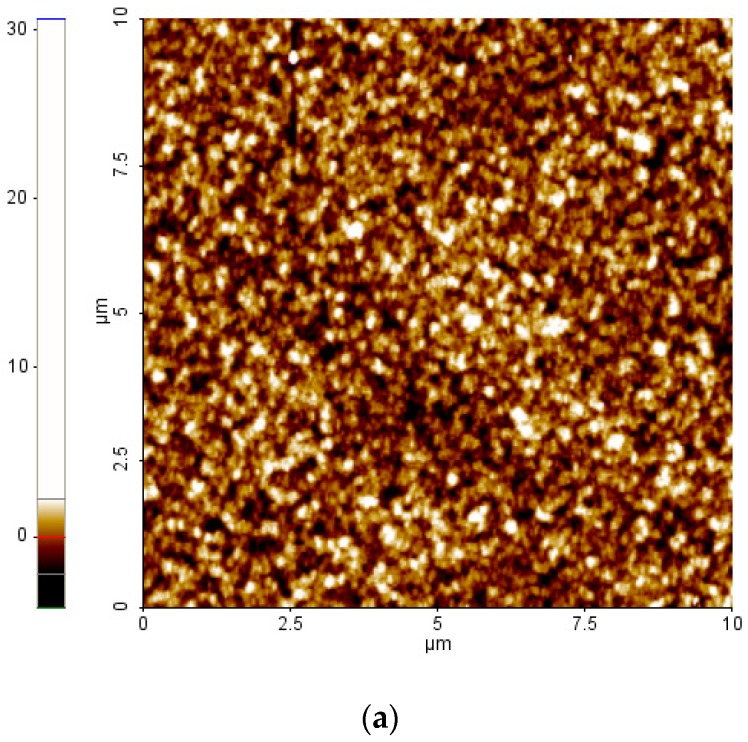

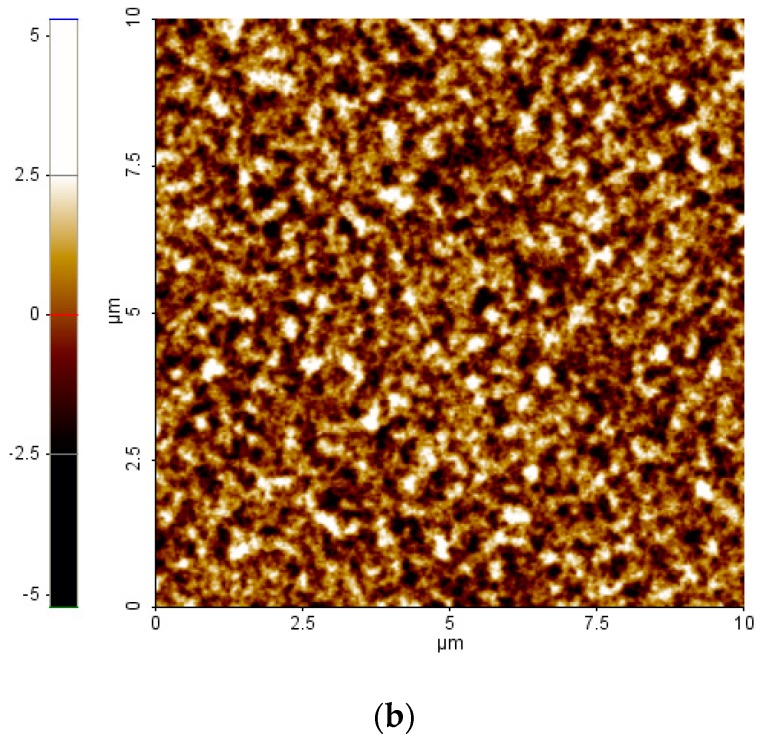
Atomic force microscopy (AFM) images of (**a**) P3HT:PCBM films and (**b**) P3HT:HTh6BT:PCBM films.

**Figure 4:**
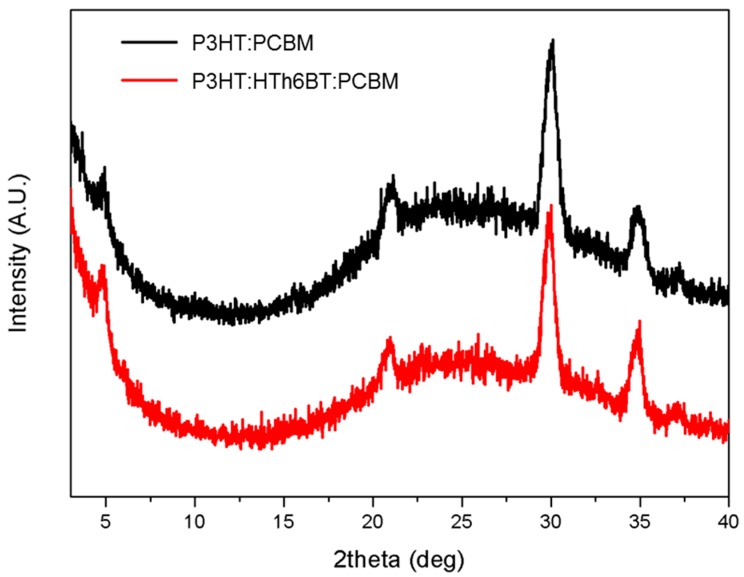
X-ray diffraction (XRD) patterns of P3HT:PCBM and P3HT:HTh6BT:PCBM films.

To measure the changes in morphology of the photoactive layer and the crystalline structure of the polymer caused by the addition of HTh6BT, atomic force microscopy (AFM) and X-ray diffraction (XRD) patterns were measured. Figure 3a,b refer to the AFM images measuring the morphology of the surfaces of P3HT:PCBM and the P3HT:HTh6BT:PCBM thin film. As HTh6BT is added, the root-mean-square roughness of the photoactive layer surface increased from 1.09 nm to 1.23 nm. This indicates an enhancement of the ordering and crystallinity by self-organization of the polymer. This enhancement in the crystallinity of the polymer thin film was confirmed by the XRD patterns shown in Figure 4. The measurement was conducted using a film manufactured with a structure of ITO/PEDOT:PSS/P3HT:PCBM or P3HT:HTh6BT:PCBM. Crystalline (100) peaks of P3HT at 2θ = 5°, and the peaks at 22°, 29°, and 34° for PEDOT:PSS, ITO, and PCBM are present, respectively. No significant differences between the XRD patterns were seen at any peaks other than at 2θ = 5°. By using Scherrer’s equation (*L* = *K*λ/*B*cos*θ*), the crystallinity for P3HT (100) was calculated. In this equation, *L* refers to the mean crystalline size, *B* is the full-width at half-maximum value of the peak, *λ* is the wavelength of the X-rays, *θ* is the reflection angle, and *K* is the Scherrer constant [9,35]. By using this equation, the crystallite size of P3HT was calculated to be 20.27 nm and 21.31 nm, without and with the introduction of HTh6BT, respectively. The increase of the crystallite size of the polymer explains the enhancement of the crystallinity of the polymer domain in the photoactive layer. The enhancement of the polymer crystallinity can influence the crystalline ordering between polymer chains and intermolecular π–π stacking, which can eventually affect the absorption, absorption range, electron transfer, and air stability of the photoactive layer. We fabricated devices of inverted structure by using optimal condition of photoactive layer to analyze air stability according to the addition of HTh6BT. As shown in Figure S3b, the device with HTh6BT additive exhibited almost constant *PCE*; on the other hand, *PCE* of the device with conventional binary blend photoactive layer decreased after 70 h. Moreover, the roughened surface by enhanced crystallinity efficiently decreases the charge-transport distance, and provides a nonscaled texture which enhances the light scattering and light absorption of the photoactive layer [9,29].

**Figure 5:**
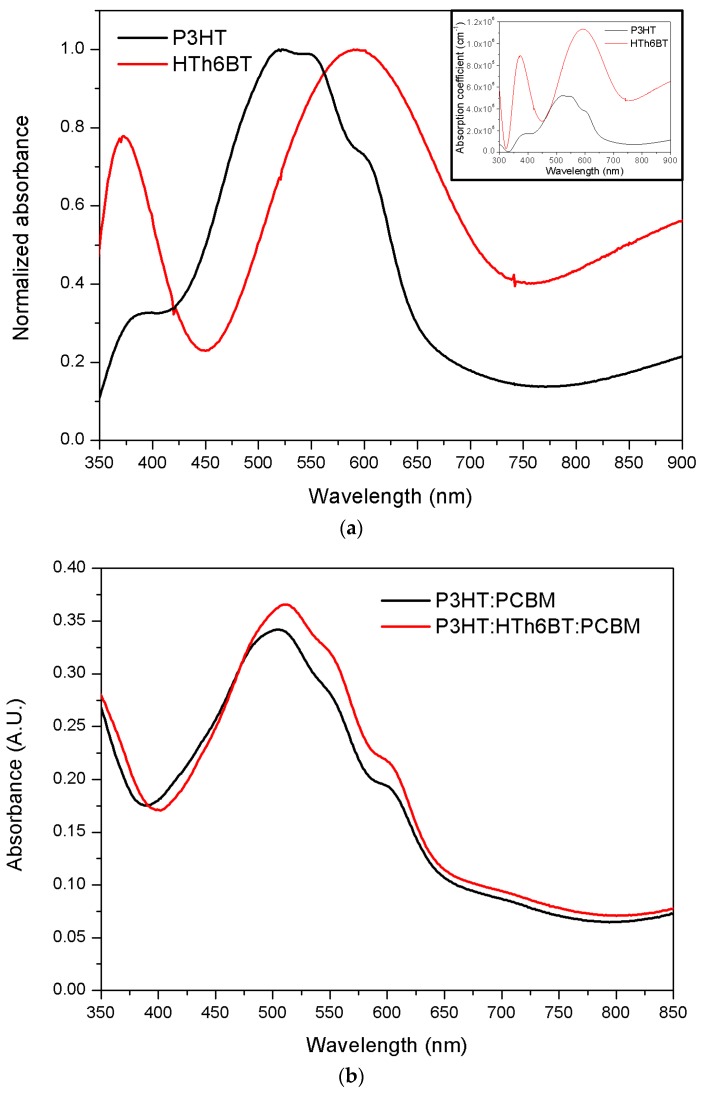
(**a**) Normalized absorbance of P3HT and HTh6BT films. (**b**) Absorbance of P3HT:PCBM and P3HT:HTh6BT:PCBM films. The inset image of (**a**) shows the absorption coefficient of the polymers.

Figure 5a shows the absorption ranges of P3HT and HTh6BT, along with the absorption coefficient, *ε*. The absorption coefficient was calculated using the equation: ε=A·ln10/L, where *A* is the absorbance and *L* is the film thickness [31]. The *λ_max_* and *ε* of P3HT were 519 nm and 5.22 × 10^5^ cm^−1^, respectively. The same values for HTh6BT were 590 nm and 1.13 × 10^6^ cm^−1^, respectively. The HTh6BT polymer with low band gap properties has a wider absorption range and higher absorption capability. Figure 5b shows the UV-Vis absorption spectra of a 160-nm-thick P3HT:PCBM film and a P3HT:HTh6BT:PCBM film. As shown in Figure 5b, the absorption increases when HTh6BT, which possesses an excellent absorption capability, is added to the P3HT:PCBM, and the absorption spectrum shows a redshift (*λ_max_* = 505 nm → 511 nm). In addition, a shoulder peak is observed at 552 nm. Such results are due to enhanced intermolecular π–π stacking and crystalline ordering caused by a strong intermolecular interaction between polymer chains [19,29,36].

**Figure 6:**
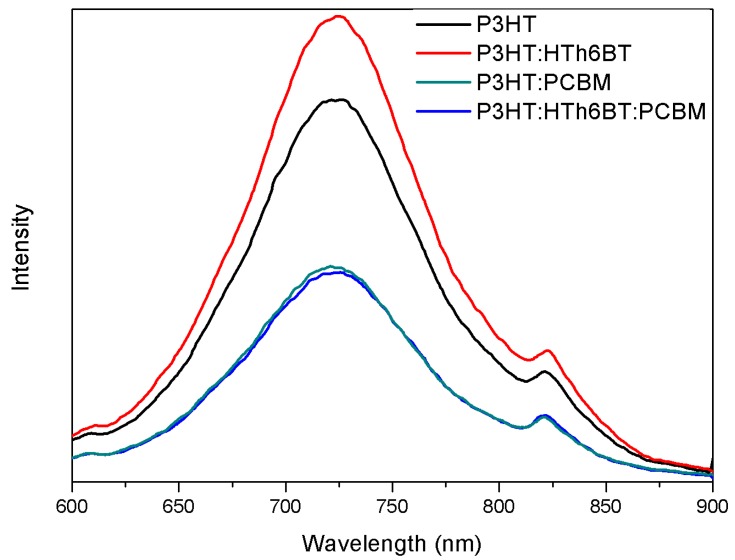
PL spectrum of P3HT, P3HT:PCBM, P3HT:HTh6BT, and P3HT:HTh6BT:PCBM films.

Figure 6 shows the PL quenching data. The area difference between the PL spectrum of the thin film without the addition of PCBM and that with the addition of PCBM indicates a quenched electron-hole. As the quenching increases, the dissociation efficiency of the electron-hole increases [9]. The quenching ratio is 44.1%, without any addition of HTh6BT. When HTh6BT is added, the quenching ratio is enhanced by 54.3%. In particular, the addition of HTh6BT triggers an even larger difference in the PL spectrum when compared to the PL spectrum difference after PCBM is added. This means that there is an increased formation of excitons in the polymer thin film as HTh6BT is added. According to this, not only the P3HT/PCBM interface, but also the HTh6BT/PCBM interface creates efficient charge dissociation. Especially, the charge generation caused by charge dissociation on the HTh6BT/PCBM interface means a contribution of enhanced *V_oc_* of the PBHJ device, as can be confirmed through the PL spectrum and PL quenching data. In addition, by swiftly transferring the charge generated by the crystallinity of the enhanced P3HT to the external circuits, and thereby suppressing the recombination of the charge, a shunt resistance (*R_sh_*) of the device is enhanced. Such enhancement leads to the enhancement of the *FF*. Consequently, an addition of HTh6BT triggers increased *J_sc_*, *V_oc_*, and *FF* values, which leads to the enhancement of the *PCE*.

## 3. Materials and Methods

HTh6BT was synthesized according to a previously reported method [3]. The photovoltaic performance of OPVs was characterized in the structure of ITO/PEDOT:PSS/photoactive layer/Al. Patterned ITO glasses were sequentially washed by sonication in detergent, 2-propanol, and deionized water. After cleaning, the ITO glasses were dried at 120 °C and ultraviolet/ozone-treated for 10 min. A thin layer (~40 nm) of PEDOT:PSS (P VP AI 4083, Heraeus, Germany) was formed by spin-coating on the cleaned ITO surface, and the substrates were baked at 140 °C for 10 min. Subsequently, the substrates were transferred into a glove box filled with nitrogen. For the photoactive layer coating, P3HT, HTh6BT, and PC_61_BM were blended at an optimal weight ratio of 1:0.2:1. The blended solution was spin-coated to form a photoactive layer of approximately 150 nm. Finally, Al (100 nm) was deposited by thermal evaporation in a high-vacuum chamber (<10^−7^ Torr). The active area of the fabricated OPVs was 4 mm^2^. The current density–voltage (*J*–*V*) characteristics of the fabricated OPVs were assessed using a Keithley 2400 source measure unit and an AM 1.5G solar simulator (Oriel 96000 150 W solar simulator, Newport corporation, Irvine, CA, USA). The incident photon-to-current conversion was measured to determine the best performance of the inverted devices.

## 4. Conclusions

In this study, by using the HTh6BT polymer, which possesses low-band gap properties and π–π stacking similar to that of P3HT, a PBHJ OPV that can easily enhance the efficiency of the device was investigated. By adding HTh6BT, the polymer crystallinity of the photoactive layer was enhanced, thereby enhancing the light absorption and electric charge transfer of the photoactive layer. The enhancements were confirmed through AFM, XRD, and UV-Vis spectra. Through the enhanced PL quenching, the generation of efficient charge dissociation at the photoactive layer was confirmed. The above results indicate the successful production of PBHJ OPVs with a 36% increase in the *PCE*, resulting from the enhancements in *J_sc_*, *V_oc_*, and *FF*.

## Figures and Tables

**Table 1:** Photovoltaic performances of organic photovoltaic (OPV) devices.

Photoactive Layer	*J_sc_* (mA/cm^2^)	*V_oc_* (V)	*FF* (%)	**PCE** (%)	*R_s_* (Ω·cm^2^)	*R_sh_* (Ω·cm^2^)
P3HT:PCBM	7.1	0.596	52.9	2.2	9.02	2300
	(6.9)	(0.596)	(49.9)	(2.02)		
HTh6BT:PCBM [5]	5.5	0.820	34.6	1.6	-	-
P3HT:HTh6BT:PCBM	7.6	0.636	62.3	3.0	7.50	3340
	(7.5)	(0.636)	(59.8)	(2.85)		

The values in the parentheses represent the average values of four cells.

## Supplementary Materials

The following are available online at www.mdpi.com/1996-1944/9/12/996/s1. Figure S1: *J–V* curves of OPV devices with different ratio of HTh6BT; Figure S2: *J–V* curves of OPV devices with optimal ratio and different annealing temperature; Figure S3: Variation of normalized *PCE* of devices, which were stored in air with constant temperature and humidity (23 °C, 50%); Table S1: Photovoltaic performances of OPV devices with different ratio of HTh6BT; Table S2: Photovoltaic performances of OPV devices with optimal ratio and different annealing temperature.
