# Supplementary material for: Enhanced Photovoltaic Properties of Bulk Heterojunction Organic Photovoltaic Devices by an Addition of a Low Band Gap Conjugated Polymer
Source: Materials (Basel). 2016 Dec 8;9(12):996. doi: 10.3390/ma9120996 (PMC5456957; doi:10.3390/ma9120996)
Supplement: Supplementary file 1 [file materials-09-00996-s001.pdf]

Supplementary Materials: Enhanced Photovoltaic Properties of Bulk Heterojunction Organic Photovoltaic Devices by an Addition of a Low Band Gap Conjugated Polymer

Eui Jin Lee, Min Hee Choi and Doo Kyung Moon

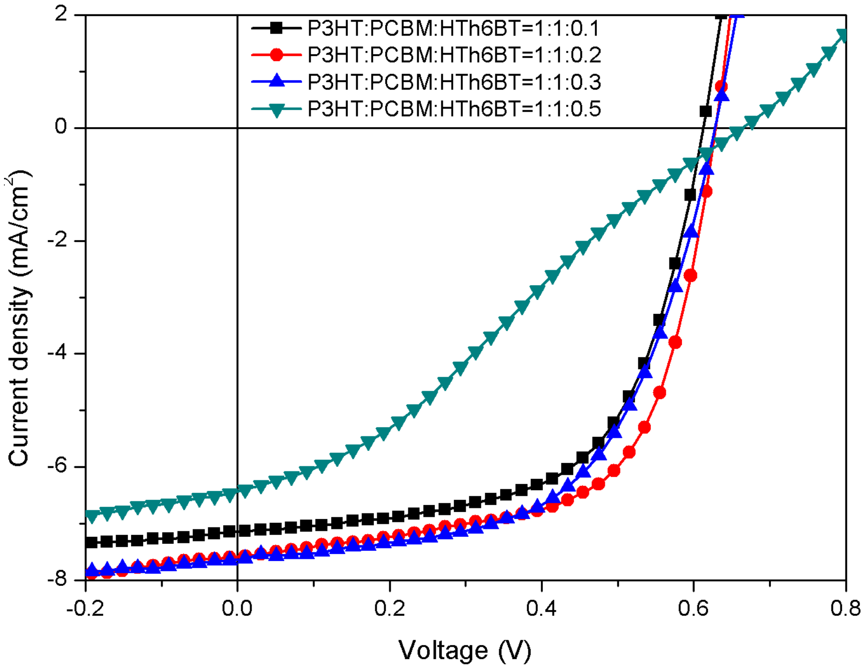

Figure S1. *J*–*V* curves of OPV devices with different ratio of HTh6BT.

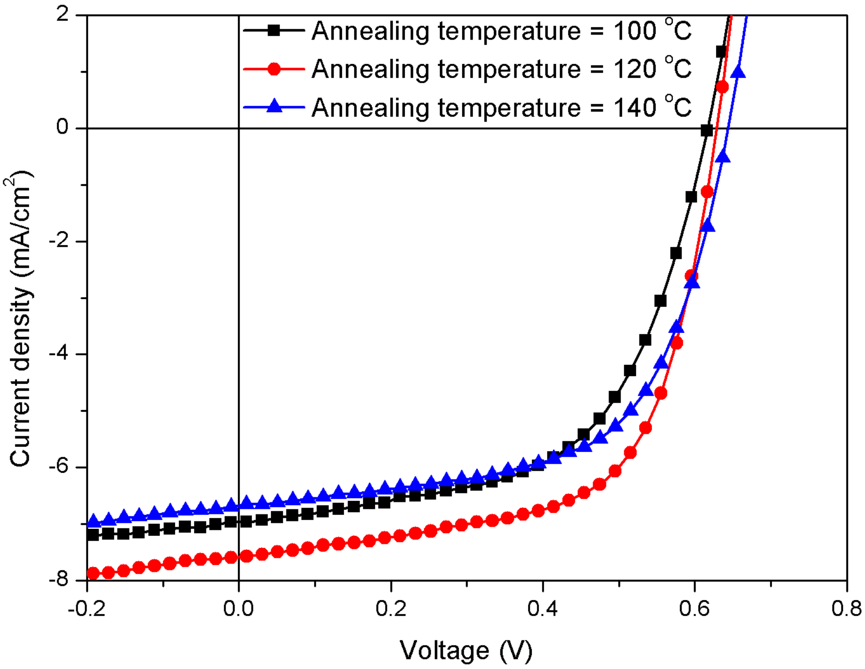

Figure S2. *J*–*V* curves of OPV devices with optimal ratio and different annealing temperature.

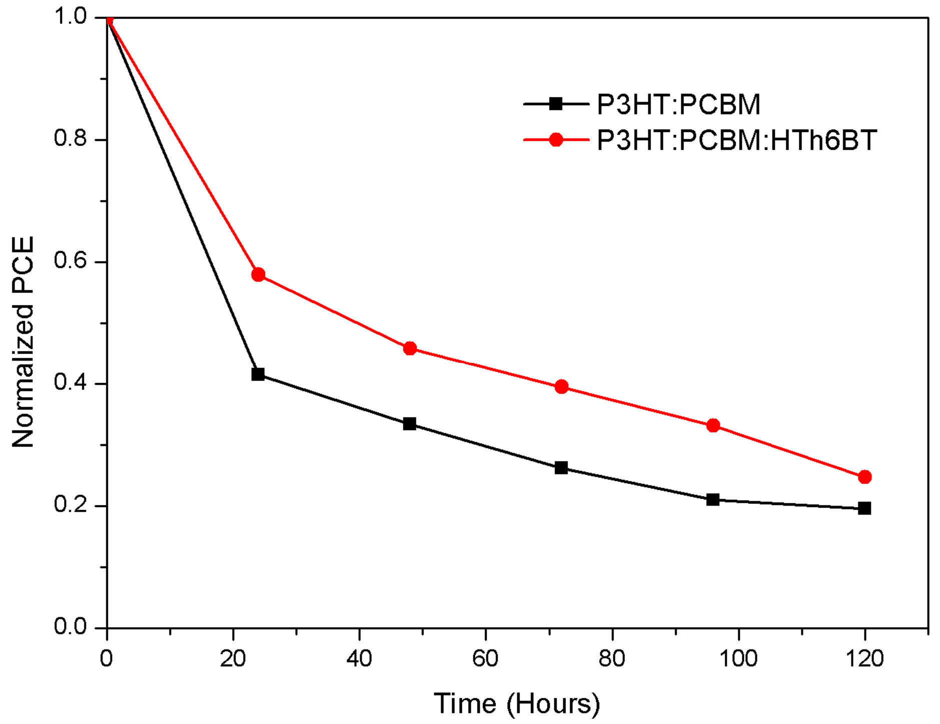

(**a**)

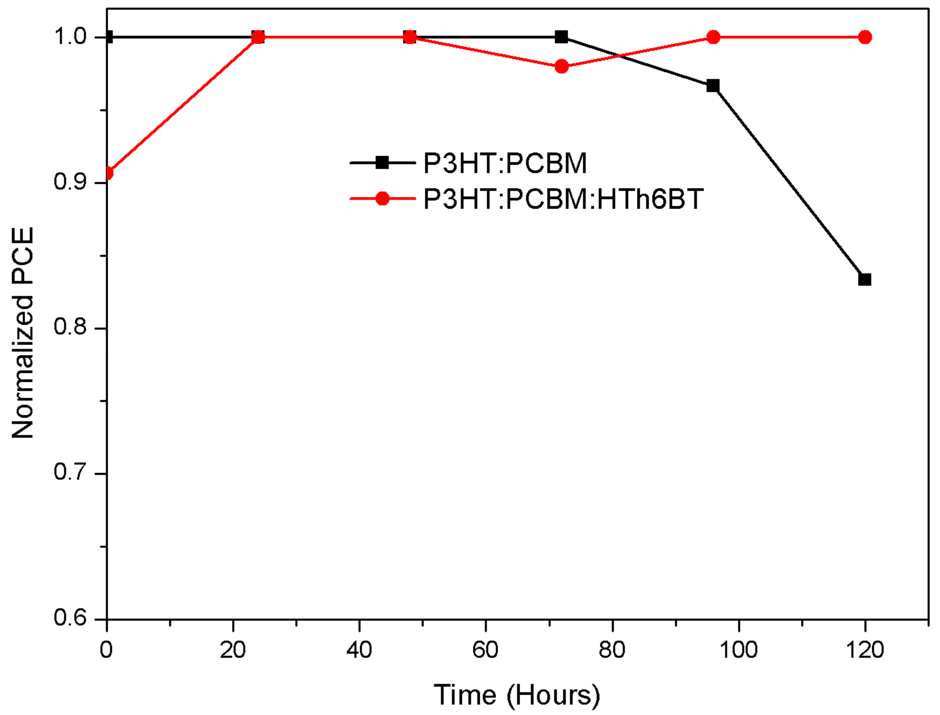

(**b**)

Figure S3. Variation of normalized PCE of devices, which were stored in air with constant temperature and humidity (23 °C, 50%) (**a**) ITO/PEDOT:PSS/Photoactive layer/Al and (**b**) ITO/ZnO/Photoactive layer/MoO_3/Ag.

Table S1. Photovoltaic performances of OPV devices with different ratio of HTh6BT.

P3HT:PCBM:HTh6BT (w/w)	J_{sc} (mA/cm²)	V_{oc} (V)	*FF* (%)	*PCE* (%)
1:1:0.1	7.1	0.616	60.4	2.7
1:1:0.2	7.6	0.636	62.3	3.0
1:1:0.3	7.6	0.636	57.2	2.8
1:1:0.5	6.4	0.657	29.5	1.2

Table S2. Photovoltaic performances of OPV devices with optimal ratio and different annealing temperature.

Annealing Temperature (°C)	J_{sc} (mA/cm²)	V_{oc} (V)	*FF* (%)	*PCE* (%)
100	7.0	0.616	57.5	2.5
120	7.6	0.636	62.3	3.0
140	6.7	0.636	61.8	2.6
